# Targeted muscle reinnervation after traumatic or elective upper-extremity amputation: a systematic review and meta-analysis

**DOI:** 10.3389/fneur.2026.1946094

**Published:** 2026-10-13

**Authors:** Fan-Long Meng, Ji-Ling Li, Xin Xue, Jia-Jia Wu, Jie Ma, Yu-Xin Zheng, Mou-Xiong Zheng, Jian-Guang Xu

**Affiliations:** 1 Department of Orthopedics and Traumatology, Shanghai Shuguang Hospital Affiliated to Shanghai University of Traditional Chinese Medicine, Shanghai, China; 2 Department of Rehabilitation Medicine, Shanghai University of Traditional Chinese Medicine Yueyang Hospital of Integrated Traditional Chinese and Western Medicine, Shanghai, China; 3 Engineering Research Center for TCM Intelligent Rehabilitation, Ministry of Education, Shanghai University of Traditional Chinese Medicine, Shanghai, China

**Keywords:** brachial plexus injury, functional outcomes, myoelectric prosthesis, targeted muscle reinnervation, upper extremity amputation

## Abstract

**Objective:**

To systematically evaluate functional recovery and prosthetic control following targeted muscle reinnervation (TMR) or TMR-based reconstruction in patients with upper extremity amputation or severe brachial plexus injury.

**Methods:**

PubMed, Embase, Web of Science, Scopus, and the Cochrane Library were searched for studies reporting standardized functional outcomes before and after TMR. Two reviewers independently performed study selection, data extraction, and methodological quality assessment using the Methodological Index for Non-Randomized Studies (MINORS). Because most studies did not report the standard deviation of paired changes or within-participant correlations, preoperative and postoperative summary data were synthesized using mean differences (MDs) or standardized mean differences (SMDs) as exploratory estimates. Potentially overlapping cohorts were identified by comparing study centers, recruitment periods, authorship, and participant characteristics.

**Results:**

Six eligible reports representing five independent patient cohorts were included. MINORS scores ranged from 9 to 12 out of 16, indicating important methodological limitations. Postoperative Southampton Hand Assessment Procedure (SHAP) scores improved significantly (MD = 31.45, 95% CI 6.69–56.21), although substantial heterogeneity was observed (*I*^2^ = 83%). Disabilities of the Arm, Shoulder and Hand (DASH) scores showed a significant reduction in self-reported disability after direction harmonization (MD = 21.91, 95% CI 10.85–32.97; *I*^2^ = 0%). A single study reported a significant improvement in Action Research Arm Test (ARAT) scores (MD = 22.00, 95% CI 10.29–33.71), while another reported an increase in OPUS upper-extremity function scores (mean change = 2.7, 95% CI 2.3–3.2). Sensitivity analysis including combined TMR and osseointegration produced a similar DASH effect.

**Conclusion:**

Current evidence suggests that TMR-based reconstruction may improve prosthetic task performance and perceived upper-limb function. However, the findings remain preliminary because of small samples, heterogeneous procedures, incomplete paired-data reporting, and the frequent use of TMR within multicomponent reconstructive programmes. Larger prospective multicenter studies using standardized functional outcomes are required.

**Systematic review registration:**

https://www.crd.york.ac.uk/PROSPERO/view/CRD420251002627, identifier (CRD420251002627).

## Introduction

Upper-extremity amputation causes substantial loss of motor function and may markedly restrict independence in activities of daily living, occupational participation, and social interaction (1–3). In addition to individuals with traumatic limb loss, some patients with severe traumatic brachial plexus injury (TBPI) may undergo elective amputation of a chronically paralysed and non-functional upper limb as part of a bionic reconstruction strategy. In these patients, amputation is not performed as an isolated intervention but forms part of a staged reconstructive pathway that may include targeted muscle reinnervation (TMR), prosthetic fitting, and structured rehabilitation. Both populations face comparable barriers to daily function and prosthetic/orthotic use. Although advances in prosthetic design have increased the mechanical capabilities and degrees of freedom of artificial limbs, effective intuitive control of these devices remains challenging across both patient groups (4, 5). Conventional myoelectric prostheses typically rely on electromyographic signals recorded from a limited number of residual muscles (6, 7). In individuals with proximal amputations (transhumeral amputation and shoulder disarticulation) and complete pan-brachial plexus injuries, available myoelectric signal sources may be insufficiently selective to support independent, simultaneous control of multiple prosthetic joints (8). As a result, patients may experience delayed responses, sequential rather than coordinated multi-joint movements, a high cognitive burden during device operation, and limited integration of prosthetic equipment into routine daily activities (9, 10).

TMR (11), introduced by Kuiken and Dumanian in 2002, was initially developed to improve the neural interface between residual peripheral nerves and externally powered upper-limb prostheses (12–15). The procedure involves transferring residual nerves that previously controlled the missing limb to motor branches of denervated or functionally expendable recipient muscles (16). Following axonal regeneration and muscle reinnervation (17), the reinnervated muscles serve as biological amplifiers of motor commands originally intended for the impaired distal upper limb (18, 19). For example, the median, ulnar, and radial nerves may be redirected to separate muscle targets, thereby creating spatially distinct electromyographic signals corresponding to different intended hand, wrist, or elbow movements (20).

The physiological basis of TMR involves both peripheral nerve regeneration and long-term central sensorimotor adaptation, consistent across amputee and brachial plexus injury populations (21). Regenerating axons establish functional connections with new muscle targets, while the central nervous system learns to use the resulting myoelectric signals for prosthetic control (22). When combined with pattern-recognition algorithms, TMR may allow the identification of multiple movement intentions from complex electromyographic activation patterns (23, 24). This neural reconstruction strategy delivers more intuitive, accurate, and multidimensional motor control compared with conventional direct myoelectric control. Enhanced signal differentiation theoretically translates to faster task completion, smoother coordinated movement, more consistent daily prosthetic wear, and superior overall functional independence for both patient groups.

Despite these potential advantages, functional recovery after TMR is not uniform. Outcomes may be influenced by the level and cause of amputation, timing of surgery, duration of denervation, condition of the residual nerves and recipient muscles, surgical technique, postoperative rehabilitation, and the characteristics of the prosthetic control system (25–27). Considerable methodological heterogeneity also exists among published studies. Motor recovery has been assessed using diverse outcomes, including electromyographic signal quality, movement-classification accuracy, prosthetic control tests, task-completion time, functional performance scales, and patient-reported measures. Differences in study design, prosthetic technology, training protocols, and follow-up duration further limit direct comparison across studies.

Therefore, a focused synthesis of the available evidence is required to determine whether TMR-based reconstruction translates into clinically meaningful improvements in upper-limb function and prosthetic control. This systematic review focuses on individuals with upper-extremity amputation who underwent TMR or TMR-based functional reconstruction. The study population includes individuals with traumatic amputation and selected patients with severe TBPI who subsequently underwent elective amputation as part of a bionic reconstruction programme. The primary objective was to evaluate postoperative motor function, prosthetic task performance, and validated measures of upper-extremity function, while also identifying factors that may contribute to variability in functional recovery. This review also aims to explore factors linked to variable functional outcomes after TMR, and to summarize shortcomings of existing upper-limb function assessment tools in relevant patients.

## Materials and methods

### Search strategy

This meta-analysis was prospectively registered in the International Prospective Register of Systematic Reviews (PROSPERO: CRD420251002627) to ensure transparency and minimize reporting bias. The implementation strictly followed the latest PRISMA guidelines (28). A systematic literature search was conducted across five electronic databases—PubMed, Embase, Web of Science, Scopus, and the Cochrane Library—from their inception through July 2026. The search strategy combined Medical Subject Headings (MeSH) and free-text terms related to “targeted muscle reinnervation” or “TMR,” alongside “amputation “or “TBPI.” The detailed query was adapted for the specific syntaxes of each database. Specific search terms are shown in Table 1.

**Table 1:** Search strategies used in each electronic database.

Electronic database	Search strategies
PubMed	(“targeted muscle reinnervation”[tiab] OR “targeted reinnervation”[tiab] OR “targeted reinnervation”[tiab] OR “targeted muscle re-innervation”[tiab] OR “targeted motor re-innervation”[tiab] OR TMR[tiab]) AND ((((“Amputees”[Mesh] OR “Amputation, Traumatic”[Mesh] OR “Amputation, Surgical”[Mesh]) AND (“Upper Extremity”[Mesh] OR “Arm”[Mesh] OR “Forearm”[Mesh] OR “Hand”[Mesh])) OR ((“upper limb”[tiab] OR “upper limbs”[tiab] OR “upper extremity”[tiab] OR “upper extremities”[tiab] OR arm[tiab] OR forearm*[tiab] OR hand*[tiab]) AND (amput*[tiab] OR amputee*[tiab] OR disarticulat*[tiab] OR “limb loss”[tiab])) OR transhumeral[tiab] OR “trans-humeral”[tiab] OR transradial[tiab] OR “trans-radial”[tiab] OR “shoulder disarticulation”[tiab] OR “elbow disarticulation”[tiab] OR “wrist disarticulation”[tiab] OR “partial hand amputation”[tiab] OR “forequarter amputation”[tiab]) OR (“Brachial Plexus Neuropathies”[Mesh] OR “brachial plexus injury”[tiab] OR “brachial plexus injuries”[tiab] OR “traumatic brachial plexus injury”[tiab] OR “adult brachial plexus injury”[tiab] OR “brachial plexus palsy”[tiab] OR “brachial plexus avulsion”[tiab] OR “brachial plexus root avulsion”[tiab] OR “global brachial plexus injury”[tiab] OR “complete brachial plexus injury”[tiab] OR “total brachial plexus injury”[tiab]))
Web of science	TS = (“targeted muscle reinnervation” OR “targeted motor reinnervation” OR “targeted reinnervation” OR “targeted muscle re-innervation” OR “targeted motor re-innervation” OR TMR) AND TS = (((“upper limb” OR “upper limbs” OR “upper extremity” OR “upper extremities” OR arm OR forearm* OR hand*) AND (amput* OR amputee* OR disarticulat* OR “limb loss”)) OR transhumeral OR “trans-humeral” OR transradial OR “trans-radial” OR “shoulder disarticulation” OR “elbow disarticulation” OR “wrist disarticulation” OR “partial hand amputation” OR “forequarter amputation” OR (“brachial plexus” AND (injur* OR trauma* OR pals* OR paralys* OR avuls*)) OR “brachial plexus injury” OR “traumatic brachial plexus injury” OR “adult brachial plexus injury” OR “brachial plexus avulsion” OR “brachial plexus root avulsion” OR “global brachial plexus injury” OR “complete brachial plexus injury” OR “total brachial plexus injury”)
Embase	(‘targeted muscle reinnervation’:ti,ab,kw OR ‘targeted motor reinnervation’:ti,ab,kw OR ‘targeted muscular reinnervation’:ti,ab,kw OR ‘targeted reinnervation’:ti,ab,kw OR ‘targeted muscle re-innervation’:ti,ab,kw OR ‘targeted motor re-innervation’:ti,ab,kw OR tmr:ti,ab,kw) AND ((‘amputation’/exp OR ‘amputation’ OR ‘amputee’/exp OR ‘amputee’ OR ‘traumatic amputation’/exp OR ‘traumatic amputation’) AND (‘upper limb’/exp OR ‘upper limb’) OR ((‘upper limb’:ti,ab,kw OR ‘upper limbs’:ti,ab,kw OR ‘upper extremity’:ti,ab,kw OR ‘upper extremities’:ti,ab,kw OR arm:ti,ab,kw OR forearm:ti,ab,kw OR hand:ti,ab,kw OR transhumeral:ti,ab,kw OR ‘trans-humeral’:ti,ab,kw OR transradial:ti,ab,kw OR ‘trans-radial’:ti,ab,kw OR ‘shoulder disarticulation’:ti,ab,kw OR ‘elbow disarticulation’:ti,ab,kw OR ‘wrist disarticulation’:ti,ab,kw OR ‘partial hand amputation’:ti,ab,kw OR ‘forequarter amputation’:ti,ab,kw) AND (amput*:ti,ab,kw OR amputee*:ti,ab,kw OR disarticulat*:ti,ab,kw OR ‘limb loss’:ti,ab,kw OR ‘residual limb’:ti,ab,kw)) OR ‘brachial plexus injury’/exp OR ‘brachial plexus injury’ OR ‘brachial plexus neuropathy’/exp OR ‘brachial plexus neuropathy’ OR ‘brachial plexus injury’:ti,ab,kw OR ‘brachial plexus injuries’:ti,ab,kw OR ‘traumatic brachial plexus injury’:ti,ab,kw OR ‘adult brachial plexus injury’:ti,ab,kw OR ‘brachial plexus palsy’:ti,ab,kw OR ‘brachial plexus avulsion’:ti,ab,kw OR ‘brachial plexus root avulsion’:ti,ab,kw OR ‘global brachial plexus injury’:ti,ab,kw OR ‘complete brachial plexus injury’:ti,ab,kw OR ‘total brachial plexus injury’:ti,ab,kw OR ‘pan plexus injury’:ti,ab,kw OR ‘pan-plexus injury’:ti,ab,kw)
Scopus	(((“Targeted Muscle Reinnervation”)) OR TMR) AND “Upper Extremity” OR mh “Amputation” OR mh “Brachial Plexus Neuropathy” OR “upper limb” OR “upper extremity” OR arm OR hand OR amputee OR amputation OR “brachial plexus”
Cochrane	(“targeted muscle reinnervation” OR TMR) AND (Amputees OR Amputation Traumatic OR Amputation Surgica) AND (Upper Extremity OR Arm OR Forearm OR Hand OR upper limb OR upper limbs OR upper extremity OR upper extremities OR arm OR forearm OR hand) AND (amput OR amputee OR disarticulat OR limb loss OR transhumeral ORtrans-humeral OR transradial OR trans-radial OR shoulder disarticulation OR elbow disarticulation OR wrist disarticulation OR partial hand amputation OR forequarter amputation) OR (Brachial Plexus Neuropathies OR brachial plexus injury OR brachial plexus injuries OR traumatic brachial plexus injury OR adult brachial plexus injury OR brachial plexus palsy OR brachial plexus avulsion OR brachial plexus root avulsion OR global brachial plexus injury OR complete brachial plexus injury OR total brachial plexus injury)

To guarantee methodological rigor, this systematic review was reported following PRISMA guidelines, and its methodological quality was appraised via A Measurement Tool to Assess Systematic Reviews-2. Rigorous procedures including prospective protocol registration, multi-database literature retrieval, and dual independent screening and data extraction were fully implemented in this study.

### Inclusion and exclusion criteria

The Population, Intervention, Comparator, Outcome, and Study design (PICOS) framework was applied to define the eligibility criteria. The eligible population consisted exclusively of patients who underwent upper-extremity amputation and TMR-based prosthetic reconstruction. Two clinical subgroups were included: (1) patients with traumatic upper-extremity amputation who underwent TMR; and (2) patients with functionally irrecoverable TBPI who underwent elective amputation of the flaccid limb, with TMR or related nerve/muscle transfers performed as part of a multistage bionic reconstruction programme. Patients with TBPI who underwent TMR or nerve transfer without subsequent amputation and prosthetic reconstruction were excluded. TMR was defined as a surgical nerve transfer procedure in which an injured or transected peripheral nerve was transferred to a residual muscle target to restore motor signals and improve neuromuscular control. Studies evaluating TMR as part of a broader reconstructive strategy were also considered when the functional contribution of TMR could be identified.

Eligible comparisons included preoperative versus postoperative functional status within the same patient cohort or comparisons between TMR and an appropriate control intervention. The primary outcomes were postoperative changes in upper extremity motor function, prosthetic control, task performance, and activities of daily living assessed using validated functional measures. For studies reporting the same outcome measure, mean differences were calculated. When different but conceptually comparable functional measures were used, standardized mean differences were applied. Studies reporting only pain relief, electromyographic signal quality, pattern-recognition accuracy, imaging findings, or technical prosthetic performance without clinically relevant functional outcomes were not included in the quantitative synthesis.

Randomized and non-randomized clinical studies were eligible, including prospective and retrospective cohort studies and case series. Studies were included in the meta-analysis when they reported sufficient quantitative data to calculate an effect estimate. Case reports and studies with relevant clinical findings but insufficient data for pooling were considered only for the qualitative synthesis. Animal studies, cadaveric studies, lower extremity amputation studies, technical studies without clinical functional outcomes, and studies lacking extractable quantitative data were excluded. Reviews, protocols, editorials, conference abstracts, grey literature, and non-peer-reviewed manuscripts were also excluded. No restrictions were applied regarding publication year or language.

Study selection was conducted independently by two reviewers. Titles and abstracts were screened first, followed by full-text assessment of potentially eligible studies. Disagreements were resolved through discussion or consultation with a third reviewer. When multiple publications reported overlapping patient populations, only the most recent, most comprehensive, or longest-follow-up study was included in the quantitative analysis, whereas earlier reports were used only to supplement methodological or clinical information.

### Data extraction

Two reviewers independently extracted data using a standardized form, including study characteristics, patient demographics, injury type, amputation level, TMR procedure, sample size, follow-up duration, functional outcome measures, and preoperative and postoperative data. For continuous outcomes, means, standard deviations, and sample sizes were recorded. For Miller et al. (29), numerical values were digitized from the published figure using Automeris.io because tabulated means and standard deviations were unavailable. Data extraction from the figure was independently checked by two reviewers, and discrepancies were resolved by consensus.

### Statistical analysis

Statistical analyses were performed using Review Manager (RevMan, version 5.3; The Cochrane Collaboration). All functional outcomes were continuous variables. As the primary comparison involved baseline and follow-up functional assessments within the same cohort, effect estimates were calculated using the time points reported in the original studies. Where baseline represented pre-TMR prosthetic performance, this was explicitly distinguished from pre-amputation function. Mean differences (MDs) with 95% confidence intervals (CIs) were calculated when studies used the same outcome measure, whereas standardized mean differences (SMDs) were used when different but conceptually comparable scales were reported. The direction of each scale was harmonized so that positive estimates consistently indicated functional improvement. A random-effects model was used because clinical and methodological heterogeneity was anticipated across studies. Statistical heterogeneity was assessed using Cochran’s Q test and the *I*^2^ statistic. Sensitivity analyses were conducted by sequentially excluding individual studies. When sufficient data were available, subgroup analyses were performed according to the clinical pathway leading to upper-extremity amputation: traumatic amputation or elective amputation for functionally irrecoverable TBPI. Statistical significance was defined as a two-sided *p* value of <0.05. Publication bias was not formally assessed because fewer than 10 studies were included in each pooled analysis.

## Results

### Study selection

The database search identified 956 records. After removing 399 duplicates, 557 records underwent title and abstract screening, of which 526 were excluded. Thirty-one reports were subsequently assessed in detail. Six reports met the eligibility criteria for quantitative synthesis. However, two reports potentially included overlapping patient populations. Thus, the six eligible reports represented five independent patient cohorts, and five independent cohorts were included in the primary quantitative synthesis. The study selection process is shown in Figure 1.

**Figure 1:**
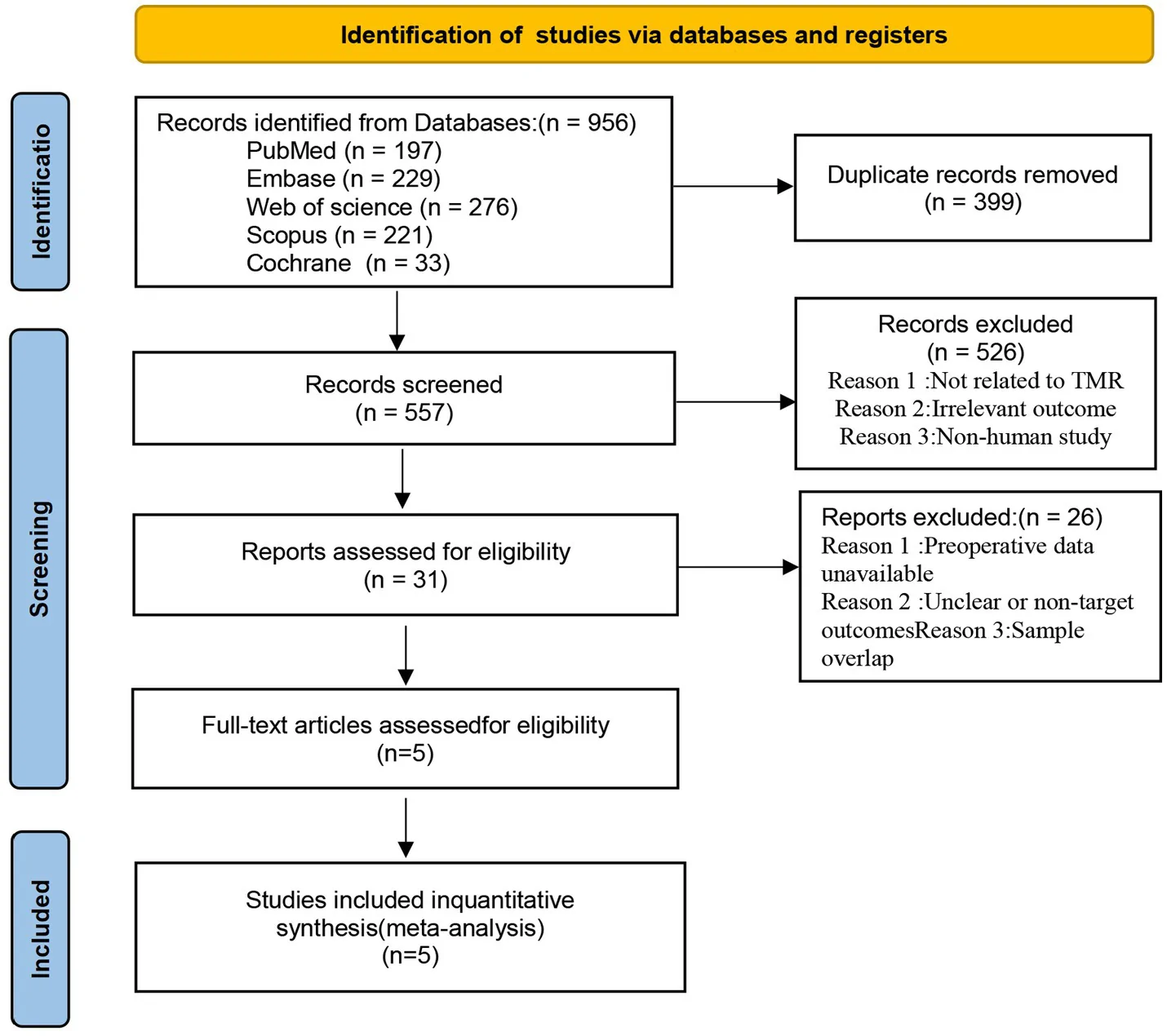
PRISMA flow diagram of the study selection process.

#### Characteristics of included studies

Six reports published between 2008 and 2023 met the eligibility criteria for quantitative synthesis, with sample sizes ranging from 3 to 33 participants. These studies were conducted primarily in the United States and Austria and included patients undergoing TMR-based prosthetic reconstruction after traumatic upper-extremity amputation or elective amputation for functionally irrecoverable TBPI. However, two reports from the same research group potentially included overlapping patient populations. Therefore, the six eligible reports were considered to represent five independent cohorts in the primary analysis. Three additional studies with relevant quantitative data were included only in separate or sensitivity analyses because of differences in study design, intervention components, or outcome reporting. Detailed study characteristics are presented in Table 2.

**Table 2:** Study characteristics.

Study	Year	Sample size ^(*n*)^	Outcome measure	Clinical population and amputation level	Baseline^h^		Follow-up^h^	
					Mean	SD	Mean	SD
Miller et al. (29)^a^	2008	6	Modified Box and Blocks test (120-s protocol)	Three transhumeral amputations and three cases treated functionally at the shoulder-disarticulation level (two shoulder disarticulations and one humeral-neck transhumeral amputation)	5.85	2.25	16.62	3.02
Simon et al. (23)^b^	2023	7	Box and Blocks test	Unilateral traumatic transradial amputation	35.90	2.60	48.00	4.30
Mioton et al. (31)^c^	2020	20	OPUS upper-extremity Rasch score	Upper-limb amputation above the wrist; specific levels not reported	53.70	3.40	56.40	3.70
Hruby et al. (32)^d^	2017	5	SHAP	Global brachial plexus injury with a nonfunctional hand; elective transradial amputation during bionic reconstruction	10.00	1.58	55.00	19.71
Hruby et al. (32)^e^	2020	3	SHAP	Severe brachial plexus avulsion; final reconstruction levels included transhumeral amputation or shoulder disarticulation	2.33	4.04	22.00	9.17
Hruby et al. (32)^d^	2017	5	ARAT	Global brachial plexus injury with a nonfunctional hand; elective transradial amputation during bionic reconstruction	3.40	4.28	25.40	12.66
Hruby et al. (32)^d^	2017	5	DASHf	Global brachial plexus injury with a nonfunctional hand; elective transradial amputation during bionic reconstruction Severe brachial plexus avulsion; final reconstruction levels	57.90	20.57	32.00	28.57
Hruby et al. (32)^d^	2020	5	DASHf	included elbow disarticulation, transhumeral amputation, or shoulder disarticulation	52.50	9.36	31.18	9.75
Sturma et al. (37)^d,g^	2024	3	DASHf (sensitivity analysis)	Traumatic transhumeral amputation; combined TMR and osseointegration	40.97	19.02	23.87	13.36

Patients with TBPI included in this review underwent elective amputation of the functionally irrecoverable limb as part of a multistage bionic reconstruction programme. Values are those used in the quantitative analyses; n denotes the number of participants included in the corresponding outcome analysis. ARAT, Action Research Arm Test; DASH, Disabilities of the Arm, Shoulder and Hand; OPUS, Orthotics and Prosthetics Users’ Survey; SD, standard deviation; SHAP, Southampton Hand Assessment Procedure; TMR, targeted muscle reinnervation.

^a^Miller et al.: Individual pre- and post-intervention values were digitized from Figure 2 using Automeris.io. Means and sample SDs were calculated from these approximate values because numerical summary statistics were not reported.

^b^Simon et al.: Eleven participants were enrolled; eight completed both pre- and post-TMR home trials, and one of these eight was excluded from the quantitative analysis, leaving seven participants.

^c^Mioton et al.: The OPUS analysis was reported with *n* = 20 in Table 4, whereas the baseline characteristics section listed 19 upper-limb amputations; the outcome-specific sample size was used.

^d^Hruby et al. (2017, 2020) and Sturma et al. (2024): Means and sample SDs were calculated from reported participant-level data; small diferences from values rounded in the source articles may therefore occur.

^e^Hruby et al. (2020): Final postoperative SHAP scores were available for three of the five participants.

^f^Lower DASH scores indicate less disability. Original DASH scores are presented in the table; values were multiplied by _1 only in the forest plots to ensure that positive effect estimates consistently represented improvement.

^g^Sturma et al. evaluated combined TMR and osseointegration and was included only in the DASH sensitivity analysis.

^h^Baseline and follow-up refer to the assessment time points reported in the original studies and do not necessarily represent pre-amputation and post-amputation status. For studies involving secondary TMR, baseline generally represents post-amputation status before TMR; for bionic reconstruction after severe TBPI, baseline represents functional status before completion of the reconstructive programme.

#### Quality assessment

Six eligible reports representing five independent patient cohorts were assessed for methodological quality using the Methodological Index for Non-Randomized Studies (MINORS) (30). All eligible reports were non-randomized and non-comparative. The tool evaluates eight domains: a clearly stated aim, consecutive patient inclusion, prospective data collection, appropriate endpoints, unbiased outcome assessment, an appropriate follow-up period, loss to follow-up of less than 5%, and prospective calculation of study size. Each item was scored as 0 if not reported, 1 if reported but inadequate, and 2 if reported and adequate, yielding a maximum score of 16, with higher scores indicating better methodological quality. Overall, the included studies reported clearly defined objectives, clinically relevant functional outcomes, and generally adequate follow-up periods; however, the evidence was limited by small sample sizes, inadequate reporting of consecutive patient inclusion, and insufficiently described unbiased outcome assessment. Only one study reported a prospective sample size calculation. Incomplete follow-up or outcome data were also identified in several studies: 10 of 43 patients in Mioton et al. (31) lacked 1-year follow-up data, while final functional outcomes in Hruby et al. 2020 were available for only three of five patients for some measures (32). Simon et al. additionally reported that study personnel were not blinded to participants’ surgical status (23). Because the studies assessed pre-TMR and post-TMR outcomes in the same participants but did not report the standard deviations of change scores or within-participant correlation coefficients, standardized mean changes could not be calculated directly. Therefore, preoperative and postoperative summary data were analysed using standardized mean differences as an exploratory approach. Potential cohort overlap was evaluated by comparing study centers, recruitment periods, authorship, injury or amputation levels, surgical procedures, and participant characteristics. When earlier or potentially overlapping publications were identified, only the most comprehensive report with the largest sample or longest follow-up was retained to avoid double counting. Detailed MINORS assessments are presented in Table 3.

**Table 3:** Methodological quality of included studies assessed using the MINORS instrument.

Study	MINORS items								Total score
	Aim (1)	Consecutive patients (2)	Prospective data (3)	Appropriate endpoints (4)	Unbiased assessment (5)	Appropriate follow-up (6)	Loss to follow-up <5% (7)	Sample-size calculation (8)	
Miller et al., 2008 (29)	2	0	2	2	0	2	2	0	10
Simon et al., 2023 (23)	2	0	2	2	1	2	1	2	12
Mioton et al., 2020 (31)	2	1	2	2	1	2	1	0	11
Hruby et al., 2017 (34)	2	1	1	2	0	2	2	0	10
Hruby et al., 2020 (32)	2	1	1	2	0	2	1	0	9
Sturma et al., 2024 (37)	2	1	1	2	1	2	2	0	11

MINORS, Methodological Index for Non-Randomized Studies. Items were: (1) clearly stated aim; (2) inclusion of consecutive patients; (3) prospective collection of data; (4) endpoints appropriate to the aim; (5) unbiased assessment of endpoints; (6) follow-up period appropriate to the aim; (7) loss to follow-up less than 5%; and (8) prospective calculation of study size. Each item was scored as 0 (not reported or criterion not fulfilled), 1 (reported but inadequate or unclear), or 2 (reported and adequate). The maximum total score for a non-comparative study is 16.

### Meta-analysis results

This meta-analysis evaluated the effects of TMR-based prosthetic reconstruction on upper extremity functional recovery in patients with traumatic upper extremity amputation and in patients who underwent elective amputation for functionally irrecoverable TBPI. The included populations comprised individuals with traumatic upper-extremity amputation and selected patients who underwent elective amputation after longstanding severe TBPI as part of a bionic reconstruction programme. Functional outcomes were analysed separately according to the assessment instrument rather than combined into a single composite measure. The number and clinical characteristics of participants contributing to each outcome are described below and summarized in Table 2. Random-effects models were applied when substantial heterogeneity was present, whereas fixed-effects models were used when heterogeneity was low or absent. Overall, the pooled estimates favored postoperative improvement, suggesting that TMR may contribute to enhanced upper limb function. However, these findings should be interpreted cautiously because of the small sample sizes and methodological heterogeneity among the included studies.

Two studies (23, 29) involving 13 participants contributed Box and Blocks Test (33) outcomes to the quantitative analysis. Miller et al. included six participants with high-level upper-extremity amputation, whereas Simon et al. contributed seven participants with transradial amputation to the quantitative functional-outcome analysis. Because the original publication by Miller et al. did not provide numerical summary data, the relevant values were extracted from the published figure using Automeris.io. As shown in Figures 2, a fixed-effects model was applied because no statistical heterogeneity was detected between the studies (*I*^2^ = 0%, *p* = 0.90). The pooled analysis showed a significant improvement in performance after TMR compared with the pre-TMR condition (SMD = 3.52, 95% CI 2.14–4.90; *Z* = 5.00, *p* < 0.00001).

**Figure 2:**
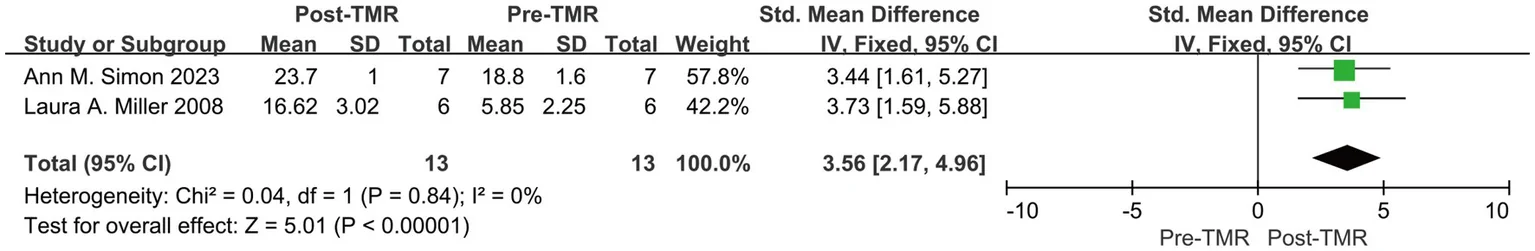
Forest plot of Box and Blocks Test outcomes before and after TMR in individuals with upper-extremity amputation.

One study (31) involving 20 individuals with upper-extremity amputation assessed patient-reported function using the OPUS Upper Extremity Rasch score. As shown in Figure 3, at 1 year after TMR, the score increased from 53.7 ± 3.4 to 56.4 ± 3.7, corresponding to a mean within-patient improvement of 2.7 points (95% CI 0.5–4.9; *Z* = 2.4; *p* = 0.02).

**Figure 3:**
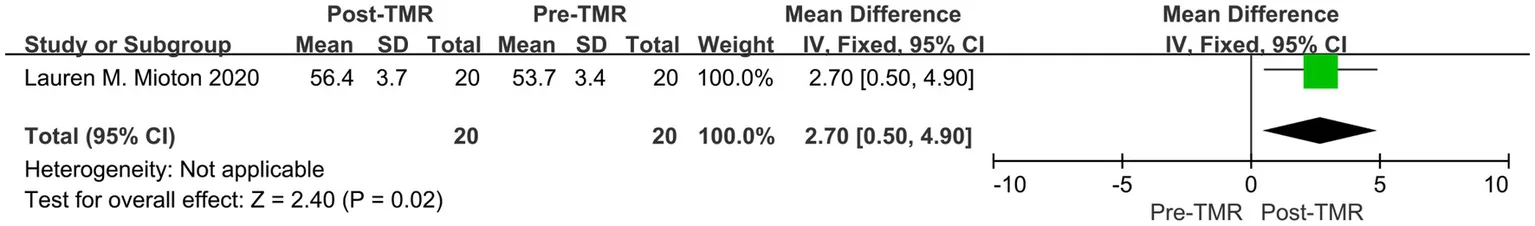
Forest plot of OPUS Upper Extremity Rasch scores before and after TM.

Two studies (32, 34) involving eight patients with longstanding severe TBPI who underwent elective amputation as part of a bionic reconstruction programme reported functional performance using the SHAP (35). As shown in Figure 4, because substantial heterogeneity was observed across the studies (*I*^2^ = 83%, *p* = 0.02), a random-effects model was applied. The pooled analysis showed that after bionic reconstruction SHAP scores were significantly higher than baseline scores (MD = 31.45, 95% CI 6.69–56.21; Z = 2.49, *p* = 0.01).

**Figure 4:**
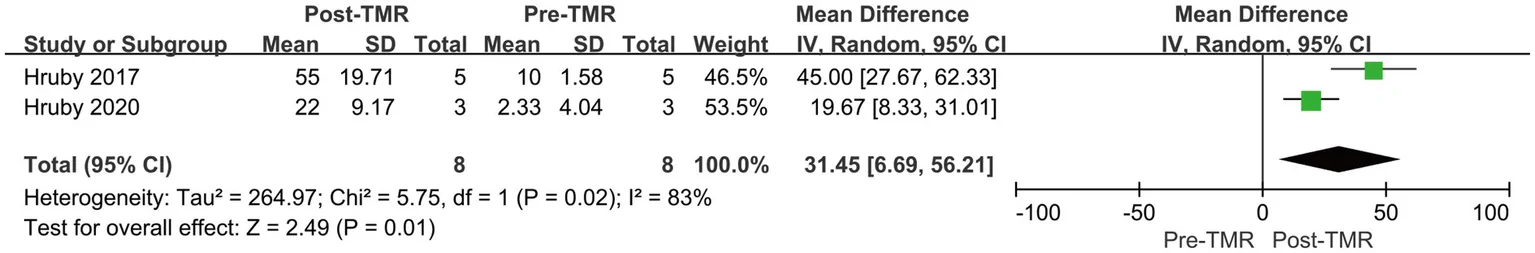
Forest plot of Southampton Hand Assessment Procedure (SHAP) scores before and after TMR-based bionic reconstruction.

One study (34) involving five patients with functionally irrecoverable TBPI who underwent elective amputation as part of TMR-based bionic reconstruction evaluated upper-extremity functional recovery using the ARAT (36). As shown in Figure 5, the mean ARAT score increased from 3.4 ± 4.28 before surgery to 25.4 ± 12.66. MD = 22.00, 95% CI 10.29–33.71; Z = 3.68, *p* = 0.0002.

**Figure 5:**
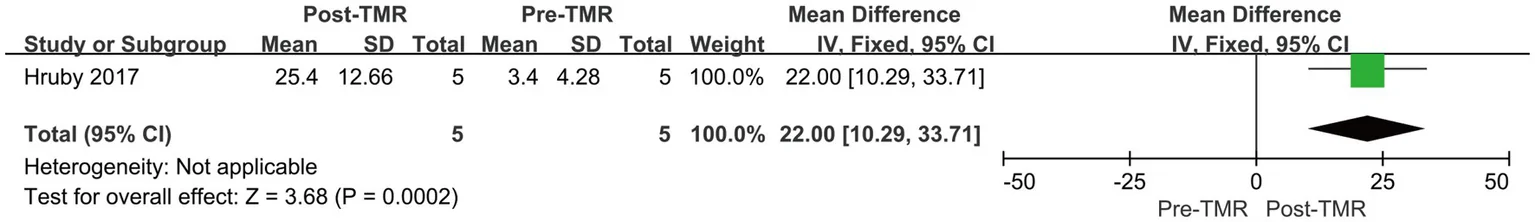
Forest plot of Action Research Arm Test (ARAT) scores before and after TMR-based bionic reconstruction.

To assess the robustness of the findings, a sensitivity analysis additionally included Sturma et al. (37), which evaluated transhumeral amputees undergoing combined TMR and osseointegration. DASH Questionnaire scores (38) were directionally reversed so that positive values consistently represented postoperative functional improvement. As shown in Figures 6, 7, in the primary analysis, the pooled mean improvement was 21.91 points (95%CI 10.85–32.97; *I*^2^ = 0%). After inclusion of the combined-intervention study, the overall effect remained statistically significant and was largely unchanged (MD = 21.19, 95% CI 10.99–31.38; *Z* = 4.07, *p* < 0.0001), with no detectable heterogeneity among the studies (*I*^2^ = 0%, *p* = 0.91). These findings support the robustness of the primary analysis. Nevertheless, because the additional study evaluated TMR in combination with osseointegration, the observed functional improvement cannot be attributed exclusively to TMR.

**Figure 6:**
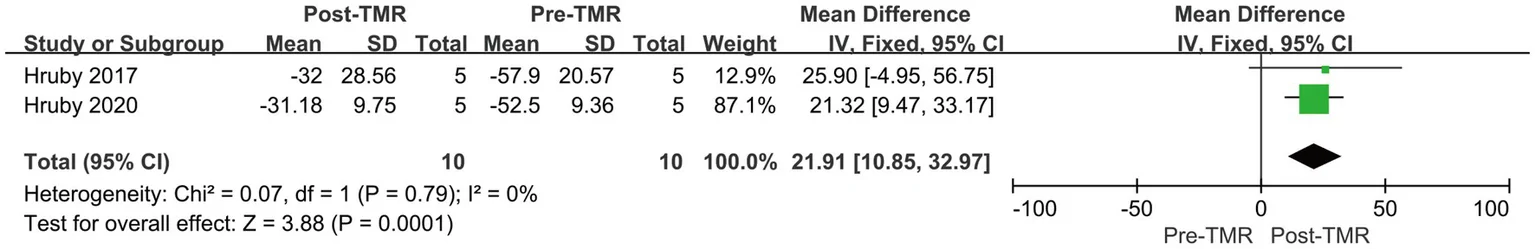
Forest plot of the primary analysis of Disabilities of the Arm, Shoulder and Hand (DASH) scores before and after TMR-based reconstruction.

**Figure 7:**
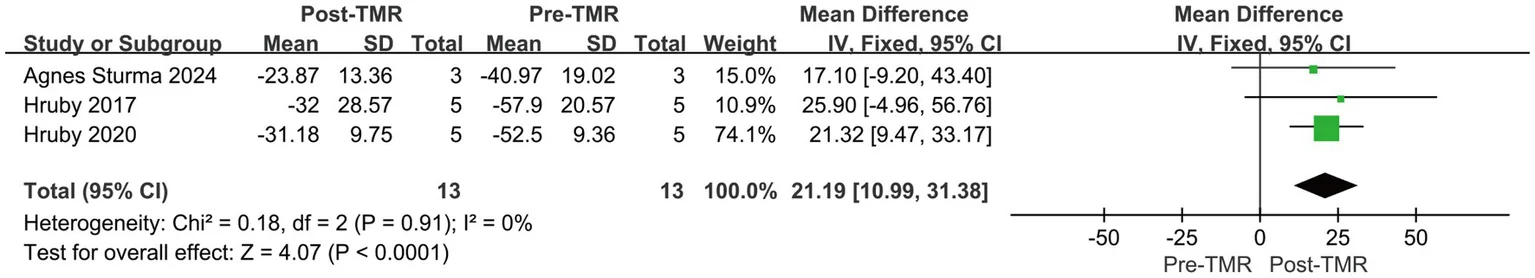
Forest plot of the sensitivity analysis of DASH scores after inclusion of the study evaluating combined TMR and osseointegration.

Given the substantial clinical heterogeneity, we conducted an exploratory subgroup analysis based on the preoperative condition: acute traumatic amputation versus chronic BPI with delayed elective amputation. This pattern likely reflects lower preoperative baseline function in the BPI group rather than a differential surgical effect.

## Discussion

This systematic review and meta-analysis suggests that TMR-based prosthetic reconstruction may improve upper-limb function in patients with traumatic upper-extremity amputation and in selected patients undergoing elective amputation for functionally irrecoverable TBPI. Improvements were observed in both performance-based assessments and patient-reported outcomes, indicating that the benefits of TMR may extend beyond the generation of additional electromyographic signals to clinically relevant gains in task performance and perceived disability. Nevertheless, the findings should be interpreted cautiously because of the small number of available studies, limited sample sizes, and substantial variation in patient populations, surgical procedures, prosthetic systems, and rehabilitation protocols.

The functional effect of TMR may differ according to the level of limb loss. In patients with proximal amputations, conventional myoelectric control is often restricted by the limited number of independent residual muscle signals. By transferring nerves from the missing limb to alternative muscle targets, TMR creates additional control sites and may restore otherwise unavailable hand and wrist motor information. This mechanism may explain the marked improvements reported in high-level amputation studies. In contrast, patients with transradial amputation retain several native forearm muscles and motor signals, and the additional benefit of TMR may therefore be smaller. Differences in amputation level may partly account for the variability in treatment effects across studies.

Clinical heterogeneity is also related to the etiology and chronicity of limb loss. Patients undergoing elective amputation after longstanding TBPI may differ substantially from those with traumatic upper extremity amputation because of prolonged denervation, muscle atrophy, previous reconstructive procedures, compensatory motor strategies, and long-term sensorimotor reorganization. These factors may influence subsequent reinnervation, prosthetic training, and functional recovery. Moreover, the intervals between the initial injury, amputation, TMR-based reconstruction, and functional assessment were inconsistently reported across studies. Therefore, outcomes from these populations should be interpreted within their specific clinical context rather than assuming equivalent baseline conditions.

The clinical value of TMR also depends on how newly generated signals are converted into prosthetic movement. Hargrove et al. (19) found that patients who had already undergone TMR achieved better performance with pattern-recognition control than with conventional direct control. Although this study was excluded from the primary meta-analysis because it evaluated the control strategy rather than the surgical effect, it helps explain the observed functional improvements. TMR should therefore be viewed as a biological interface whose effectiveness depends on signal-processing algorithms, prosthetic hardware, socket design, training, and rehabilitation rather than as an isolated procedure that automatically restores function.

This interpretation is supported by engineering and physiological studies (39). Research on electrode placement, signal separability, pattern-recognition accuracy, and prosthetic degrees of freedom has consistently shown that TMR can increase the amount of usable motor information (40). Myers et al. (41), for example, reported increases in detectable electromyographic signals and controllable prosthetic functions after surgery. Other studies using electrode arrays (42), ultrasound-based interfaces, and machine-learning methods have further demonstrated the potential of TMR to support more complex prosthetic control. These findings provide a plausible mechanistic basis for the improvements detected in clinical assessments. However, better signal classification under laboratory conditions does not necessarily translate into greater independence in daily life. Future studies should therefore combine technical outcomes with standardized functional assessments and real-world prosthesis use.

The evidence from severe brachial plexus injury requires separate interpretation. In these patients, TMR-related nerve transfers were usually incorporated into a broader bionic reconstruction pathway involving muscle transfer, electromyographic training, elective amputation, prosthetic fitting, and prolonged rehabilitation (43, 44). The resulting functional gains therefore reflect the entire treatment programme rather than TMR alone. Nevertheless, TMR remains an important component because it creates or strengthens the motor signals required for prosthetic control. For patients with a chronically paralysed, insensate, and non-functional hand after failed biological reconstruction, this approach may provide functional opportunities that cannot be achieved through conventional nerve reconstruction alone.

Reports involving free functional muscle transfer and neurotized muscle flaps also demonstrate the expanding indications for TMR. In patients with very proximal amputations or insufficient residual muscle tissue, suitable target muscles may be unavailable. Creating new biological amplifiers through transferred or neurotized muscle tissue may enable prosthetic control in otherwise unsuitable candidates. These studies are clinically important but are generally based on small case series and combined procedures, making them more appropriate for demonstrating feasibility than for estimating a generalizable treatment effect.

The early TMR literature was essential in establishing surgical feasibility and intuitive prosthetic control (13, 45–47), but several reports originated from the same specialized research centers and may have included overlapping patients. The same individuals were sometimes described in publications focusing on surgical technique, electromyographic performance, prosthetic control, or long-term function. Treating these reports as independent studies would result in double counting and overestimation of the evidence. The present review therefore prioritized the most comprehensive reports for quantitative synthesis and used earlier publications primarily to describe the development of the technique.

Some clinically relevant studies could not be pooled because they lacked preoperative functional data. Salminger et al. (48) reported postoperative outcomes in a relatively large TMR cohort, but only a subset underwent standardized functional evaluation and corresponding baseline measurements were unavailable. These data cannot provide a reliable estimate of within-patient improvement, but they remain useful for describing the level of function achievable after surgery, prosthetic fitting, and rehabilitation. This limitation highlights the need for prospective studies with standardized assessments before surgery and at predefined follow-up intervals.

The present findings have several clinical implications. Successful recovery after TMR depends on the availability of appropriate donor nerves and target muscles, sufficient time for reinnervation, optimal prosthetic fitting, and structured rehabilitation (49). Outcomes may also be influenced by amputation level, duration of denervation, previous reconstruction, prosthetic experience, and additional procedures such as osseointegration or muscle transfer. These factors should be considered when selecting patients and discussing expected outcomes.

Several limitations should be acknowledged. Most included studies were small, non-randomized case series conducted in highly specialized centers. Considerable clinical heterogeneity existed in patient characteristics, surgical techniques, prosthetic components, rehabilitation exposure, and follow-up duration. Some studies evaluated isolated TMR, whereas others assessed multicomponent bionic reconstruction (50). Paired change-score variances were not consistently reported, and some numerical data required extraction from published figures. The limited number of studies also restricted subgroup analyses and prevented reliable assessment of publication bias. The included studies encompassed two distinct clinical trajectories: patients with traumatic amputation receiving TMR at or shortly after the time of limb loss, and patients with chronic brachial plexus injury undergoing elective amputation and TMR years after the initial denervation. These populations differ profoundly in the duration of muscle denervation, cortical reorganization, pain syndromes, and preoperative functional status. Consequently, pooling their outcome data assumes a comparability that is not clinically valid. Our analyses should therefore be interpreted as preliminary, and the findings cannot be generalized equally to both groups.

Future studies should adopt prospective multicenter designs and standardize the reporting of amputation level, nerve-transfer configuration, prosthetic technology, rehabilitation protocols, and adverse events. Outcomes should be measured before surgery and at consistent postoperative time points using both objective task-based assessments and patient-reported measures. Reporting individual change scores or paired change-score variances would also improve the reliability of future meta-analyses. Comparative studies are needed to distinguish the contribution of TMR from that of advanced control algorithms, osseointegration, muscle transfer, and intensive rehabilitation.

In conclusion, current evidence supports the functional potential of TMR-based prosthetic reconstruction in patients with traumatic upper extremity amputation and in selected patients undergoing elective amputation for functionally irrecoverable TBPI. In the latter subgroup, TMR should be interpreted as one component of a multistage bionic reconstruction programme rather than as an isolated intervention. Larger and methodologically rigorous studies are required to determine its independent effect and identify the patients most likely to achieve meaningful functional improvement.

## Data Availability

Data will be made available on request.
